# Everybody's Page

**Published:** 1889-08-17

**Authors:** 


### Everybody's Page.

The best "Epsom salts" are to be obtained from the Hunyadi Janos mineral water of Hungary.

D. Nesteroosky, a Russian doctor, has found the skin of the frog the best to graft on chronic ulcers, from its freedom from glands and hairs.

One legend of the origin of the number seven being regarded as sacred is that there are seven holes in a man's head—two eyes, two ears, two nostrils, and one mouth.

The Doctor: "I think, my dear sir, that it would be better to have a consultation; your wife is seriously ill." Anxious husband: "There! I told my wife long ago she ought to have proper medical advice, but she was so afraid of hurting your feelings."

Our ancestresses had a compound called oil of talc for the complexion. According to Fuller, the talc made a kind of whitewash, which, he said, "some justify lawful, because clearing, not changing the complexion." We have heard the same plea in our own day from ladies who disapprove of the rouge, but think there is nothing objectionable in the use of powder.

Of all the simple and profitable forms of agriculture that can be followed in the tropics, cocoa cultivation in Ceylon seems to be one of the best. The plants, grown from fresh seed, need only be set about three or four feet apart, and left to take care of themselves. No machinery is required, the leaves are plucked as they reach maturity, and on good soil the average yield is 800lb. of leaf per acre. It has been calculated that the profit on a cocoa farm is 45 per cent.

In the absence of the hero of "She," who, we understand is pursuing his researches in Central Asia, some French investigators have undertaken to show the way to the fountain of perpetual youth. It is doubtful if M. Brown Sequard's methods will ever become popular; but it is highly probable that ladies who begin to perceive the envious crow's feet of age invading their countenances will visit the "Dynamodermic Institute of Paris," where, according to Dr. Vernoy, the use of electricity will remove the tell-tale wrinkles from their faces.

It has long been known that alum will clear muddy water, but an American scientist, Prof. Leeds, in using it for that purpose, discovered that it also precipitated bacteria that filled the water in the town of Mount Holly, New Jersey. In about fifteen drops of water he found bacteria enough to form 8,000 colonies of these pleasing micro-organisms. A very small quantity of alum reduced the number to 80, and these all of the larger order that could be got rid of by filtering. The amount of alum used was so small as not to affect the taste of the water, or to be injurious to the health of those who consumed it.

When diluting acids with water it is necessary to add the acid very slowly to the water, and not the water to the acid. A rather curious accident happened at a "mineral water" factory through forgetfulness of this rule. Some large jars of sulphuric acid fell from a truck and broke, the acid collecting in a pool in a gutter in front of the factory. One workmen tried to drive the acid into the sewer by pouring a stream of water into it. The result was an immediate evolution of steam so violent as almost to be an explosion, the result of the heat generated by the union of the water and the acid; and several of the bystanders were more or less injured by it.

That formation of hard skin on the hands which simulates eczema can often be relieved by rubbing in lanoline or some wool fat with pumice stone.

A Chinese bride, when putting on her wedding garments, stands in a round shallow basket. This is supposed to ensure a placid, well-rounded life in her new home.

Dr. Sonnenberger, of Worms, looks upon antipyrin as almost a specific in whooping-cough. It should be given as early in the disease as possible, its tendency being to cut short its course, reducing it to a mild bronchial catarrh; but it is useful in any stage of the ailment.

Red roses are the best for making otto of rose, being richer in the aromatic principle of essential oils, but if a certain percentage of white roses be added the oil is more easily solidified. The otto is sometimes adulterated with geranium oil, imported from Constantinople. The geranium oil is sprinkled on the rose leaves before they are distilled, and thus becomes more ultimately mixed with the oil of rose than it could be by any subsequent addition.

Erasmus was one of the first of sanitarians. Coming from the Netherlands, where cleanliness was worshipped long before the creed spread to English shores, he was utterly disgusted at the filthiness of the streets, the over-crowding of the population, and the lack of cleanliness in the general habits of the people. Although he was offered a good benefice by Cardinal Wolsey, and a pension of six hundred florins by Henry VIII., the bribe was insufficient to overcome his natural distaste for the unhealthy condition of the English capital.

Dr. Byron Bramwell, of Edinburgh, suggests that the best bribe for school-children of the poorer class would be a good meal of bread and milk rather than marks and prizes. This, he says, at once offers the most powerful means of stimulating the child to work, and gives it as the reward of its work the thing it most requires. Considering the number of children that go to school without breakfast, there is no doubt that such a prize would not only encourage, but enable them to work, but some provision would have to be made for the children whose intellect has been brought below the average by generations of semi-starvation.

The alarum clock of ordinary life is a nuisance, but a brief one. When it strikes up its melody at the hour when you ought to be getting up in the morning—and meant to do the night before—you have only to wait till the noise has ceased, turn round, and sleep the more soundly for the interruption. It is otherwise with the awakener used by the mail-carriers of Morocco. They tie a string round their foot, and before lying down to sleep set one end of it on fire. They know, by experience, that the string will burn for a certain time, and that when it reaches the foot it is time to get up. There is no evading a call of this nature.

In America an outcry has been raised, and not a day too soon, against the atrocious caligraphy of the average doctor. It does not matter much when he is writing private letters—that is, it could not matter much under such circumstances if the average doctor ever had time to write private letters, nor would it matter if he were writing a book; the printer and he could fight out the meaning of the hieroglyphics between them. But it is different when he is writing a prescription, or if the druggist cannot decipher it he must either avow his ignorance, with the result of being despised and perhaps deserted by his customer, or else imperil the patient's life by chancing the medicine he sends.

